# DALMATIAN: An Algorithm for Automatic Cell Detection and Counting in 3D

**DOI:** 10.3389/fnana.2017.00117

**Published:** 2017-12-12

**Authors:** Sergey A. Shuvaev, Alexander A. Lazutkin, Alexander V. Kedrov, Konstantin V. Anokhin, Grigori N. Enikolopov, Alexei A. Koulakov

**Affiliations:** ^1^Cold Spring Harbor Laboratory, Cold Spring Harbor, NY, United States; ^2^Brain Stem Cell Laboratory, NBIC, Moscow Institute of Physics and Technology, Moscow, Russia; ^3^Center for Developmental Genetics and Department of Anesthesiology, Stony Brook University, Stony Brook, NY, United States; ^4^P.K. Anokhin Institute of Normal Physiology, Moscow, Russia; ^5^National Research Center “Kurchatov Institute”, Moscow, Russia

**Keywords:** brain, cell, eye, molecular and cellular imaging, microscopy, quantification and estimation, segmentation, Vessels

## Abstract

Current 3D imaging methods, including optical projection tomography, light-sheet microscopy, block-face imaging, and serial two photon tomography enable visualization of large samples of biological tissue. Large volumes of data obtained at high resolution require development of automatic image processing techniques, such as algorithms for automatic cell detection or, more generally, point-like object detection. Current approaches to automated cell detection suffer from difficulties originating from detection of particular cell types, cell populations of different brightness, non-uniformly stained, and overlapping cells. In this study, we present a set of algorithms for robust automatic cell detection in 3D. Our algorithms are suitable for, but not limited to, whole brain regions and individual brain sections. We used watershed procedure to split regional maxima representing overlapping cells. We developed a bootstrap Gaussian fit procedure to evaluate the statistical significance of detected cells. We compared cell detection quality of our algorithm and other software using 42 samples, representing 6 staining and imaging techniques. The results provided by our algorithm matched manual expert quantification with signal-to-noise dependent confidence, including samples with cells of different brightness, non-uniformly stained, and overlapping cells for whole brain regions and individual tissue sections. Our algorithm provided the best cell detection quality among tested free and commercial software.

## Introduction

GROWING evidence suggests that counts of various cell types identified by gene expression, internal and external markers correlate with various important factors, including central nervous system activity (Gage et al., 2008), impact of drugs of abuse (Eisch and Harburg, 2006), disease (Jin et al., 2004; Geraerts et al., 2007), aging (Jin et al., 2003), and other conditions (Cameron et al., 1998; Gao et al., 2009; Torner et al., 2009; Tanti et al., 2012). Currently, the majority of studies are conducted using two-dimensional tissue section techniques (Peterson, 2004; Schmitz and Hof, 2005). This traditional approach, combined with planar microscopy techniques, has numerous drawbacks, including low throughput capacity (Howard and Reed, 2010), loss of data due to interpolation (West, 2012), and difficulty in recovering 3D information. Low throughput capacity usually results in quantification performed on a subset of tissue sections (Mouton, 2011). Moreover, instead of the full information about cell positions in the sample, only average cell counts may be calculated. Such estimates may lead to incorrect values, biases, and, consequently, to inaccurate results (West, 2012).

These drawbacks have resulted in the emergence of three-dimensional fluorescent microscopy techniques. Confocal microscopy, for example, enables researchers to capture thick (up to several millimeters) tissue sections with the consequent ability to directly estimate the absolute number of cells in entire structure of interest (Encinas and Enikolopov, 2008). Other imaging techniques include optical projection tomography (Sharpe et al., 2002; Fieramonti et al., 2012), light-sheet microscopy (Dodt et al., 2007; Keller and Dodt, 2012), block-face imaging (Weninger et al., 2006; Verveer et al., 2007), and serial two photon tomography (Ragan et al., 2012). Recently, new methods for whole-mount immunohistochemical staining were described (Chung et al., 2013; Gleave et al., 2013; Renier et al., 2014). Despite an interest in three-dimensional imaging of biological objects, the set of algorithms for automatic cell detection is currently limited (Bordiuk et al., 2014).

In early studies, quantification of cellular populations was directly performed by the observer. More recently, the amount of data that has become available through advances in 3D-imaging techniques has become enormous. In 3D methods, sample preparation and capturing do not require significant interaction with volume of interest, but manual quantification is nearly impossible as a result of the enormous number of objects of interest (e.g., ~30,000 dividing cells in early postnatal murine hippocampus). The need for efficient and accurate automatic cell detection and counting algorithms has become obvious. In addition, automatic quantification can help overcome other difficulties, such as significant variations between experts (up to 60%; Schmitz et al., 1999) and complex comparisons of imaging datasets across platforms.

To respond to this need, a number of software packages for 3D reconstruction and quantification has been developed (Meijering, 2012). In agreement with the earlier careful tests (Schmitz et al., 2014), our results have shown significant deviations between the results of existing algorithms and the expert quantification. Therefore, the goal of our study was to determine the specific problems involved in automatic quantification and create algorithms that would alleviate these issues. Herein, we present a software package for robust three-dimensional quantification of fluorescently labeled cell populations that solves a range of typical problems of automatic cell detection. We called our algorithm Dependable Algorithm for Matrix Image Analysis (DALMATIAN). This algorithm is suitable for, but not limited to, whole brain samples and individual tissue sections.

## Methods

We used histogram equalization to equalize 3D image data intensities in the dataset. Voxel intensities in every 3D image were changed so that the histogram of every 3D image matched the histogram of the sample in which the parameters of the algorithm were adjusted.

To suppress autofluorescence, we subtracted the autofluorescent background obtained using imaging at different wavelengths compared to data (i.e., 488 nm for background vs. 405 or 555 nm for data). The subtraction resulted in suppressing the autofluorescent background and highlighting the signal, as we equalized the 3D image histograms as described in the previous step.

To eliminate the variations in signal and background, we used Gaussian 3D high-pass and low-pass filters. The standard deviation of the Gaussian high-pass filter had to be both larger than the typical cell radius and smaller than the typical background feature radius. The best compromise value for the standard deviation of the high-pass filter typically exceeded putative cell radius by the order of 1.5–2. The Gaussian low-pass filter standard deviation had to be both larger than the typical noise artifact radius and smaller than the typical cell radius. The best compromise value for the standard deviation of the low-pass filter was typically between 0.5 and several pixels. We implemented both high-pass and low-pass filters within a single Fast Fourier Transform (FFT) procedure. To suppress the remaining background, we set all the voxels with the intensities below the given threshold, typically 5–10% of the maximal intensity, to zero. We selected this threshold not to exceed the cell signal intensity. By doing so, we eliminated the variations in signal and background.

To perform segmentation, we used the watershed procedure on the negative 3D image. The watershed procedure divides the 3D image data into disjoint 3D segments, each containing a local intensity maximum (Malpica et al., 1997). We expect each intensity maximum to represent a whole cell or the other object of the similar size, as we removed the features larger and smaller than the average cell. Thus, each segment may contain zero or one cell. In addition, we eliminate the segments substantially smaller than expected cell volume (under 10–100 voxels).

To evaluate the statistical significance of the obtained 3D segments, we applied the following bootstrap procedure to each of the 3D watershed segments. We fit the voxel intensity within each of the segments with a 3D Gaussian distribution. For the fit, we used only the voxels of each segment within a given radius from the local intensity maximum. This radius, typically exceeding the putative cell radius by a factor of 1.5, i.e., about 7 pixels for the samples of our resolution, allows including both voxels of the cell and of the background. For these voxels, we used the unfiltered intensity data for the fit.

We performed the fit using multiple (~1,000) bootstrap iterations (**Figure 4**). Each time we randomly resampled the voxel list with repetitions. We fitted the unfiltered intensities of the resampled voxel list with a Gaussian distribution.

$$\begin{array}{l} I\left(\vec{r}\right)=I_{0}\exp\left(-\frac{1}{2}{\vec{r}}^{T}{\hat{\Sigma }}^{-1}\vec{r}\right) \end{array}$$

We used a linear regression to estimate the variance matrix $\hat{\Sigma }$. Its eigenvalues correspond to the principal components of the variance (**Figure 4**). Fraction of the resamples with the principal components within the user-defined ranges determines the statistical significance of the segment.

### Test samples and parameters

To estimate the cell detection quality, we used samples of 6 different types, 7 samples per type (42 samples total). In every sample, expert annotations were available for small cutouts containing ~10–100 cells. Our comparisons with human experts were performed for these cutouts, however, cell detection was performed for the entire samples including 400–30,000 cells.

Whole mount (WM) samples of Nestin-CFPnuc (CFP) (Encinas et al., 2006) and 5-ethynil-2′-deoxyuridine (EdU) stained hippocampi were captured using a laser scanning confocal microscope Olympus FluoView 1000 with a water immersion objective (20x, 0.5NA) at the axial resolution of 7 μm and the lateral resolution of 2 μm. For these samples (CFP WM and EdU WM, 7 samples each), we used the following parameters: low (high)-pass filter standard deviations of 0.4 (10) pixels, intensity thresholds 5% (CFP WM) and 10% (EdU WM) of maximal intensity, minimal region size of 10 voxels, fitted standard deviations larger than 1.5 pixels, *p*-value 0.01.

Tissue section (50 μm) samples of 5-ethynil-2′-deoxyuridine (EdU), 5-bromo-2′-deoxyuridine (BrdU) and c-Fos stained cells were captured using a spinning disc confocal microscope Andor Revolution WD with air objective (40x, 0.95NA) at axial resolution 0.5 μm and lateral resolution 1 μm. For these samples (EdU, BrdU, and c-Fos, 7 samples each) we used the following parameters: low (high)-pass filter standard deviations of 2 (6), 2 (3), and 2 (3) pixels, intensity thresholds of 6% (EdU), 8% (BrdU) and 4% (c-Fos) of maximum intensity, minimal region size of 100 voxels, fitted standard deviations between 4 and 9 pixels, *p*-values of 0.3 (EdU), 0.5 (BrdU) and 0.1 (c-Fos). For samples of DAPI stained tissue sections (7 samples), imaging conditions and detection parameters matched those of EdU samples.

For each of 42 samples, we performed 1,000 bootstrap iterations on voxels within 7-pixel radius. As a measure of the detection quality, we used the *F*-score (Fawcett, 2006; Selinummi et al., 2009), which is the normalized harmonic mean of the precision and the recall [*F* = 2 · precision · recall/(precision + recall)]. For the ground truth, we used cell detection by a single trained human expert per sample type. Different experts analyzed different sample types. We compared the detection quality of our algorithm with that of the other software. We used FIJI (Schindelin et al., 2012), and Imaris (Bitplane Inc.). In addition, we analyzed the dependence of the detection quality on the signal-to-noise ratio (SNR). We defined SNR as 20 logarithms of the average signal amplitude to the average noise amplitude ratio. The average signal amplitude was measured as a difference between signal and background, whereas the average noise amplitude was measured as a standard deviation of the data after high-pass filtering.

## Results

### Challenges for the automatic algorithms of cell detection

**Figure 1:**
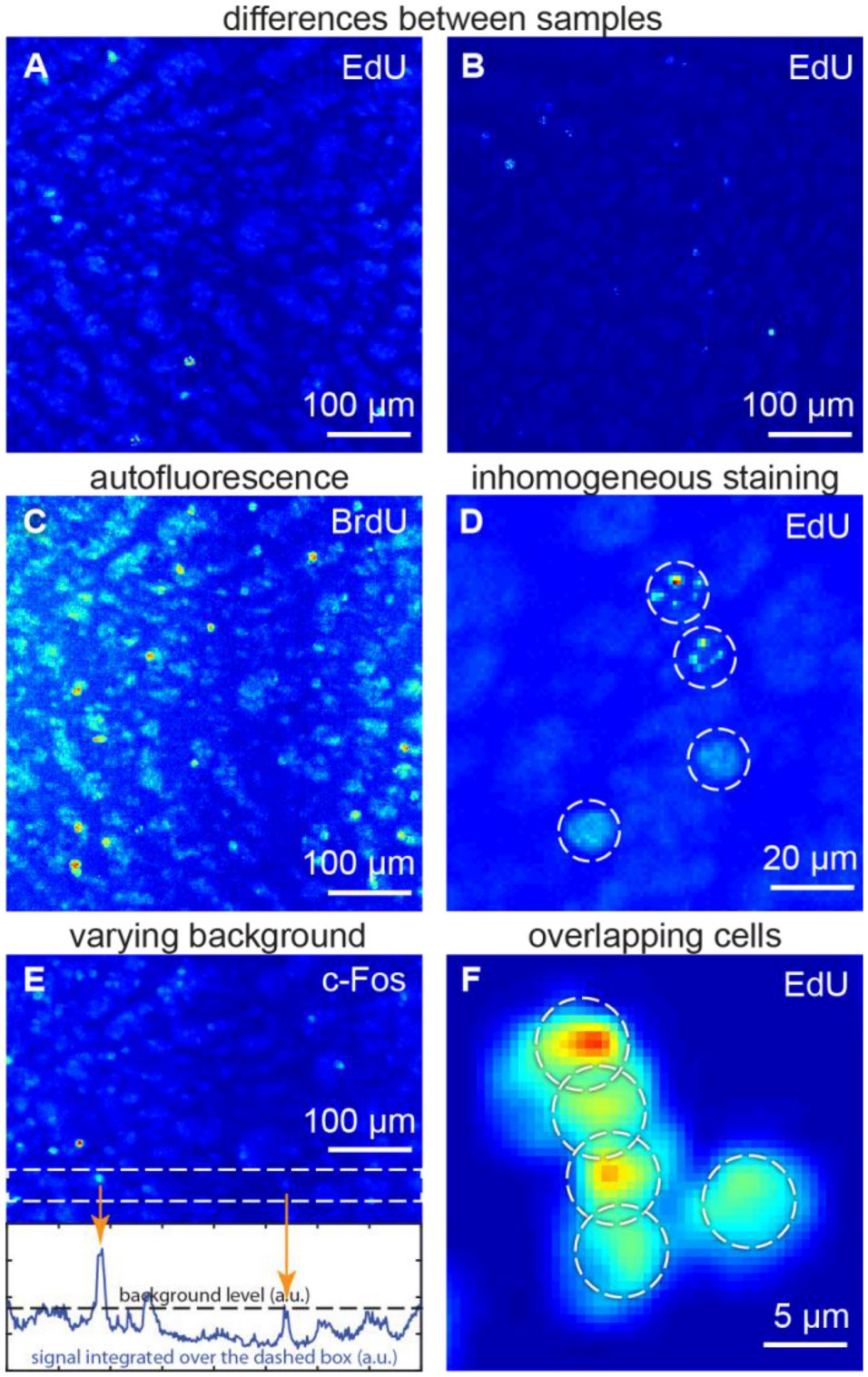
Challenges for the automatic algorithms of cell detection: **(A,B)** differences between samples, **(C)** autofluorescence, **(D)** inhomogeneous staining, **(E)** varying background, **(F)** overlapping cells. **(A,C,E)** show the same sample, thus autofluorescence patterns are repeated. All figures: maximum intensity projections of 3D images.

We focused on the following specific problems with regard to cell detection (Figure 1):

- *Differences between samples* may affect morphology, signal and background (Figures 1A,B). Therefore, tuning of parameters for each sample may be required for a typical cell detection algorithm.
- *Autofluorescence* may make the objects, which do not carry any fluorescent marker, to be as bright as the marked objects of interest (Figure 1C). Major autofluorescent molecules, such as lipofuscins, elastin and collagen, or Schiff's bases can be reduced or bleached (Viegas et al., 2007). Otherwise, both objects of interest and autofluorescent objects may contribute to cell counts, giving rise to errors (Schnell et al., 1999).
- *Inhomogeneous staining* is typical for studies of dividing cells (Figure 1D). Dividing cells are studied using synthetic thymidine analogs, which incorporate into DNA along with regular thymidine. Synthetic thymidine analogs may distribute in the cell nucleus in patches. Such nuclei may be detected as several objects or may be not detected at all (Lindeberg, 1994).
- *Varying background*. If the fluorescent background in one part of the sample is brighter than the marked cells in another parts (Figure 1E), cell count errors may also rise, as there would be no general threshold differentiating signal and background for the entire image (Otsu, 1975; Jones et al., 2005; Xiong et al., 2006).
- *Overlapping cells* (Figure 1F) may result from cellular division (which is important in proliferation studies) or may be found in samples with densely packed cells (retina, dentate gyrus etc.). Overlaps may make different cells difficult to distinguish (Malpica et al., 1997).

As each of the challenges above may result in cell counting errors, the successful algorithm is expected to address all of them.

### Our algorithm addresses differences between samples

**Figure 2:**
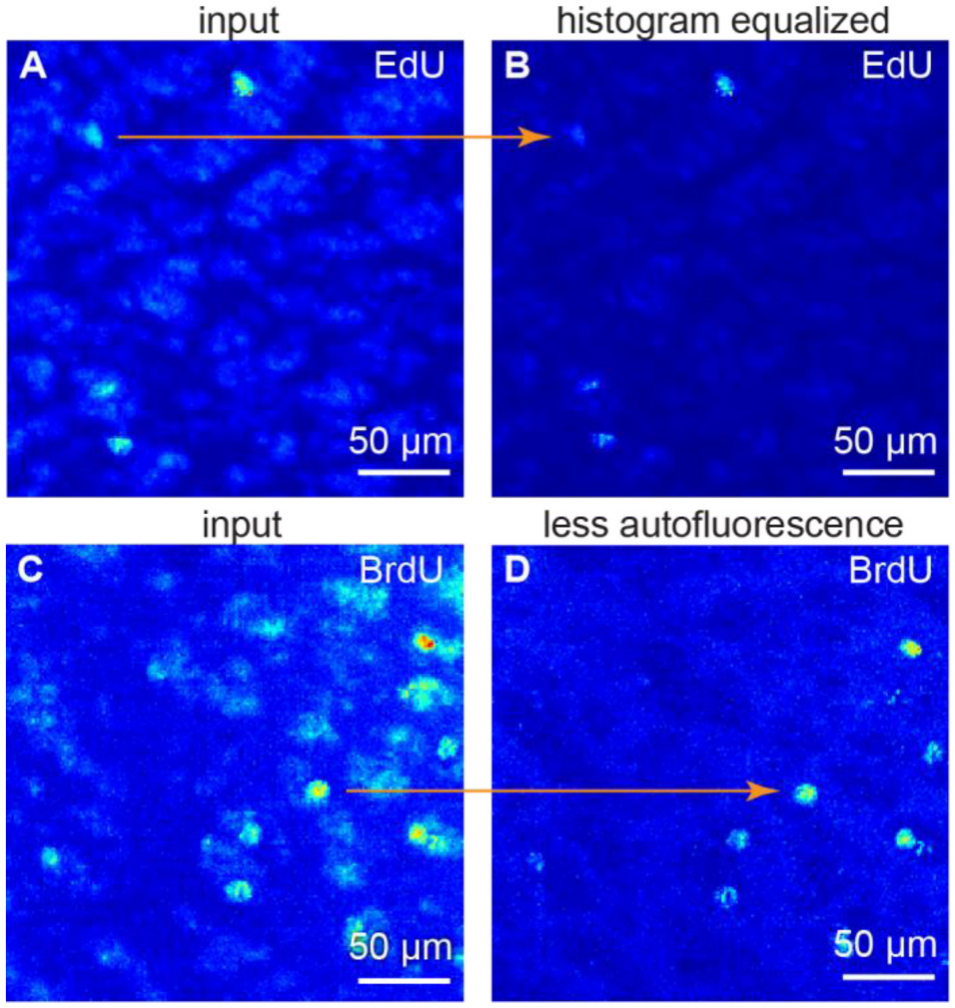
Image preprocessing. **(A,B)** histogram equalization. **(C,D)** suppressing autofluorescence. To remove autofluorescence we subtracted the images of the same sample obtained at different wavelength. All figures: maximum intensity projections of 3D images.

Fluorescence intensity relation between samples may be non-linear, as background intensity may scale separately from the signal intensity. To alleviate these differences, we use histogram equalization to make all the histograms equal in the dataset (Figures 2A,B). As a result, both background and signal intensities match among the samples. After this procedure, one can use the same set of parameters for every sample. Thus, the batch cell counting is possible.

### Our algorithm is effective in handling autofluorescence

Spectrum of autofluorescent objects (blood vessels, cells etc.) is broader than spectrum of fluorescent markers (Troy and Rice, 2004). Thus, taking the second image at a different wavelength (e.g., 488 nm as opposed to 555 nm) allows capturing autofluorescent background, but not the signal. The original and the second images, captured at a different wavelengths, may differ—a challenge identical to the previous one. Thus, we also use histogram equalization to alleviate these differences. Once the histograms are equal, the background levels match among the samples. We subtract the autofluorescent background image from the original one. As the original image is a combination of the fluorescent signal and autofluorescent background, as a result we get the signal preserved and the autofluorescence suppressed (Figures 2C,D).

### Our algorithm is resistant to inhomogeneous staining

**Figure 3:**
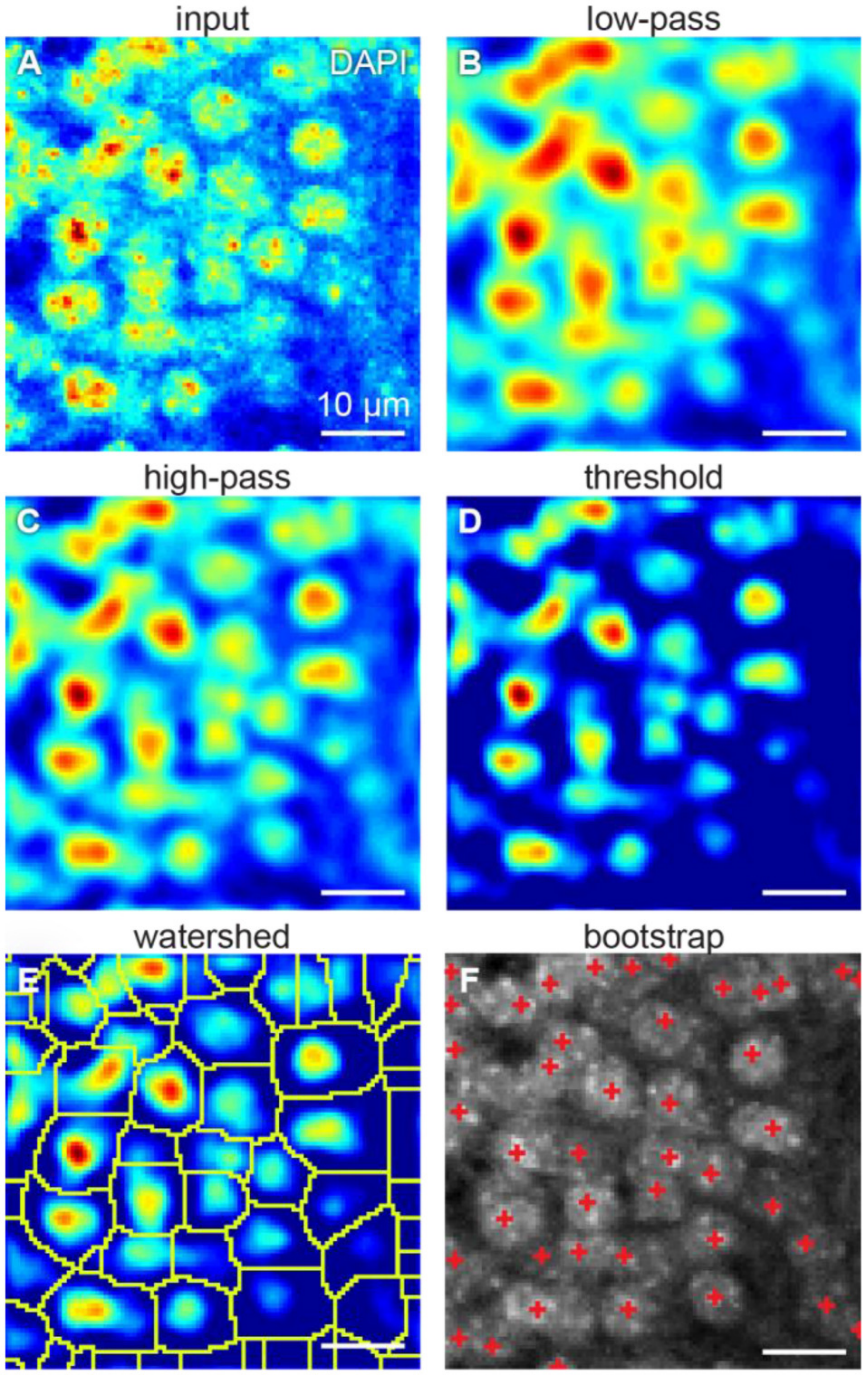
Steps of our cell detection algorithm: **(A)** original image, **(B)** Gaussian 3D low-pass, and **(C)** high-pass filters, **(D)** threshold correction, **(E)** watershed transform, and **(F)** bootstrap procedure. All figures: maximum intensity projections of 3D images.

One way to count the cells is to isolate them from each other. Cells can be isolated using fluorescent intensity minima between them. However, undesirable local intensity minima within the cells, reflecting inhomogeneous staining, may arise (Figure 3A). These minima potentially lead to splitting cells into the compartments, which may affect cell count. To overcome that challenge, we use Gaussian 3D low-pass filter. Averaging the fluorescent intensity over the region smaller than a cell, but larger than a typical staining inhomogeneity, low-pass filter removes local intensity minima within the cells, preserving those between the cells (Figure 3B). Thus, we expect inhomogeneous staining not to affect the cell isolation.

### Our algorithm is resistant to varying background

Fluorescent background gradients may also affect cell isolation. Should the cell be located on the intensity slope, it may be not separated from the others by the intensity minimum. To reduce the impact of the background, we use Gaussian 3D high-pass filter. Averaging the fluorescent intensity over the region larger than a cell provides us with an image of the fluorescent background. By subtracting that background image from the original one we equalize the intensity baseline for every cell (Figure 3C). To suppress the remaining background, we assign zero to all the voxels with fluorescent intensities below a selected threshold (Figure 3D). These steps diminish the impact of the varying background on cell isolation.

To facilitate the performance of both 3D low-pass and high-pass Gaussian filters, we applied it in frequency domain using fast Fourier transform (FFT).

### Our algorithm is successful in splitting the overlapping cells

Low-pass filtering reduces the occurrences of local intensity minima within the cells. Different cells can be separated using the intensity minima between them. To this end, we used the watershed algorithm (Figure 3E). Starting with the global intensity maximum, watershed algorithm visits all the voxels in order of the intensity decrease. If a visited voxel is a standalone, the algorithm assigns a new integer label to it. Otherwise, if a visited voxel is adjacent to a labeled one, it shares the same label. Should a visited voxel be adjacent to two voxels with distinct labels, its value will be set to zero, which means it is a boundary between cells. Thus, we divide the entire 3D volume into the segments each containing a single intensity maximum. Preprocessing with Gaussian high-pass and low-pass filters allows to reduce the creation of tiny areas. Should the tiny areas still be created, they are eliminated from the analysis based on their size. This procedure allows us to split overlapping cells.

### Our algorithm includes methods for statistical testing of the detected regions using bootstrap

**Figure 4:**
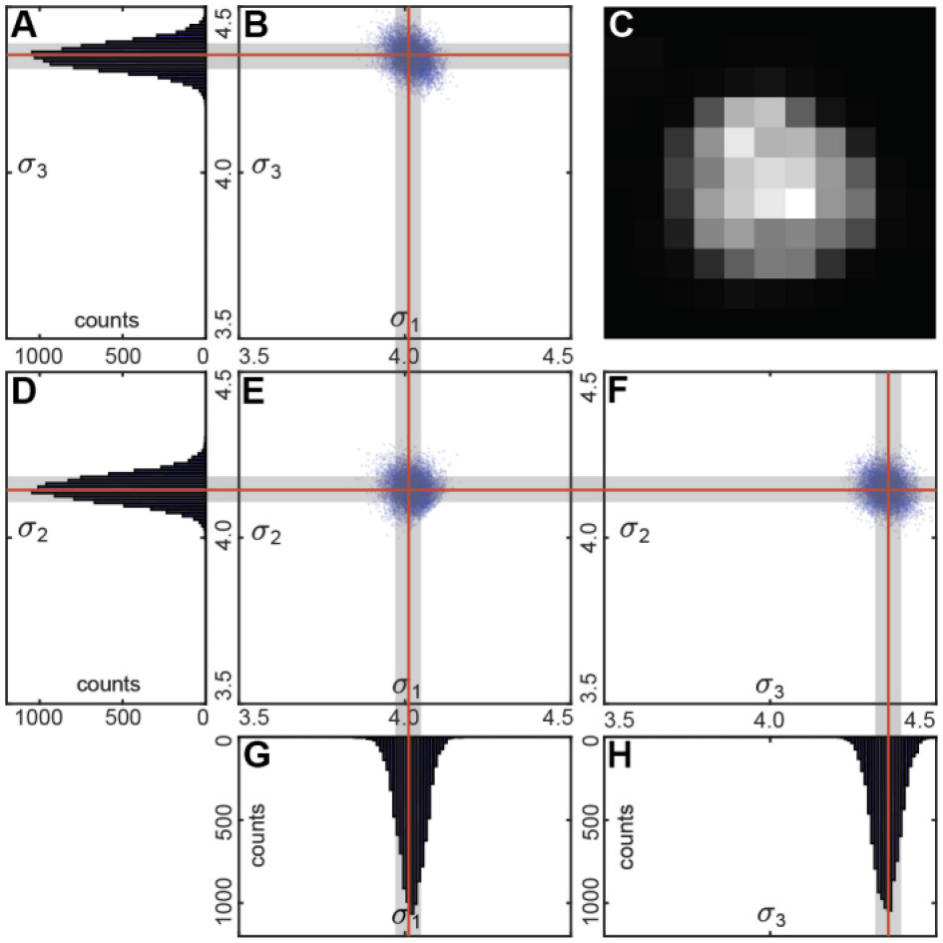
**(C)** Sample cell. Bootstrap Gaussian fit: **(B,E,F)** principal components of the standard deviation for all the resamples and **(A,D,G,H)** its histograms. Mean values are indicated by the red lines, and standard deviations by the gray boxes.

To determine, which of the segments represents a cell, we fit the intensity in every segment with a Gaussian distribution. We use the original unfiltered data for the fits, as it allows more conservative estimation of the distribution parameters. Only voxels of the segment within a selected radius from the intensity maximum are used, to reduce the impact of the background of the image. To estimate the statistical variability of the parameters of the fit, we use the bootstrap procedure (Hogg, 2005). In the bootstrap, the fits are estimated for multiple resamples of the same data (Figure 4). The resampled data is obtained from the original 3D images by selecting voxels within the same region with repetitions. Increasing the number of voxels included in fits makes the estimation of distribution of fitting parameters more accurate (Hogg, 2005). Using the distribution of fitting parameters (standard deviations and intensity) we then estimate the probability that a given segment satisfies definition of a cell provided by the user. For example, if a cell is defined as an object whose half-axes in each direction extend less than *d* voxels independently of the brightness, for each watershed segment we will compute the fraction of bootstrap iterations that do not satisfy this criterion. This fraction is interpreted as a *p*-value, i.e., the probability that a given watershed segment is not a cell. Cells are then identified as segments that yield *p*-value less than a pre-defined threshold (usually taken to be *p* = 0.05, Figure 3F).

Below, we show the results of testing of our algorithm using various samples. We compared the cell detection provided automatically by our algorithm to the cell detection produced by human experts in the same samples. We also compared our results with the commercial and free cell detection software, including FIJI (Schindelin et al., 2012) TrackMate, and Imaris (Bitplane Inc.).

### Our algorithm yields high cell detection quality for tissue sections

To show how our algorithm deals with inhomogeneous staining, we present the testing results for 50 μm thick brain sections stained with EdU and BrdU and captured in 3D using a confocal microscope (**Figures 6A,B**). EdU and BrdU, being the synthetic analogs of thymidine, incorporate into DNA of the dividing cells. Later, depending on the cell cycle phase, EdU and BrdU may distribute in the cell nucleus in patches. To estimate the cell detection quality, we use the *F*-score, which is the normalized harmonic mean of the precision and the recall [*F* = 2 · precision · recall/(precision + recall)]. To remind, precision = TP/(TP + FP), recall = TP/(TP + FN), where TP, FP, and FN are true positives, false positives and false negatives, respectively.

Cell detection quality provided by our algorithm is 90 ± 2% *F*-score for EdU and 85 ± 7% *F*-score for BrdU. Notably, the higher cell detection quality in EdU samples, compared to the one in BrdU samples, is consistent with the higher SNR: 6.2 dB and −3.6 dB, respectively. We also observed a similar trend using other software. For the same set of samples, FIJI provided cell detection quality of 86 ± 6% for EdU and 71 ± 8% for BrdU, whereas Imaris provided the quality of 85 ± 7% for EdU and 58 ± 22% for BrdU (all in *F*-scores). Our algorithm yields highest cell detection quality for these samples when compared to other software.

Similarly, to show how our algorithm deals with varying background, we used c-Fos and DAPI stained tissue section samples (**Figures 6C,D**). c-Fos features a low SNR (−6.3 dB), increasing the impact of the background on the cell detection. For our samples, SNR of DAPI images (2.81 dB) exceed that of c-Fos images. SNR of DAPI samples may be impaired due to the membrane permeability, which would also increase the impact of the background on the cell detection.

Cell detection quality provided by our algorithm equals to 86 ± 7% for c-Fos and 92 ± 3% for DAPI, which is also consistent with the expectation based on SNR. FIJI provided the cell detection quality of 81 ± 7% for c-Fos and 77 ± 6% for DAPI, whereas Imaris provided the cell detection quality of 67 ± 25% for c-Fos and 78 ± 8% for DAPI on the same set of samples (all measures are in *F*-scores). Runtime and memory usage for our algorithm matched that of both Imaris and FIJI: it took roughly 2–5 min to load and process a tissue section (roughly 1,500 × 3,000 × 30 pixels) using a laptop (Core 2 Duo ASUS laptop with 2 GB RAM). Thus, our algorithm is efficient in addressing varying background. Moreover, for the given set of samples, our algorithm provided the best level of *F*-score compared to two other software packages (FIJI and Imaris).

### Our algorithm yields high cell detection quality for whole mount samples

**Figure 5:**
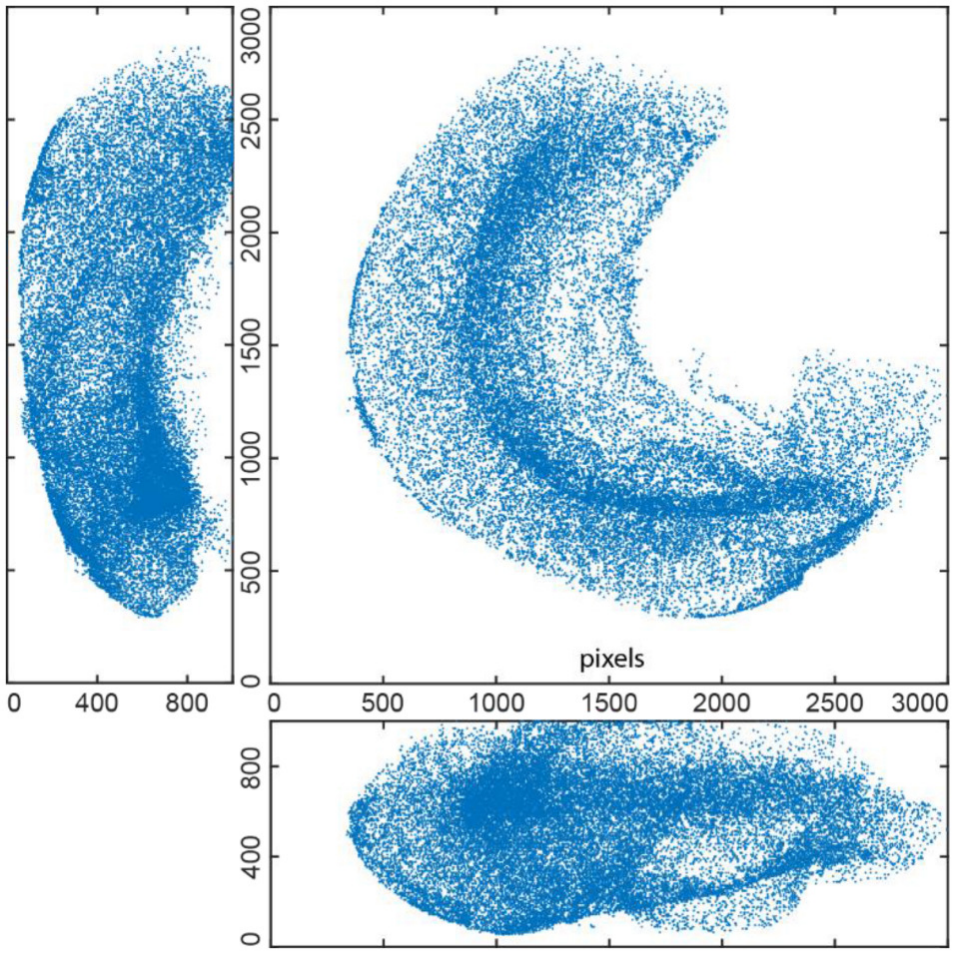
EdU+ cells detected in whole mount stained 3D hippocampus of P14 mouse (3 projections). This reconstruction includes 36451 cells.

Although both tissue sections and whole mount (WM) samples are in 3D, there are important differences between them. Sample volume is larger in WM samples (Figure 5), which may impose memory constraints. Consequently, resolution in WM samples is usually coarser than in sections to make the amount of the data manageable. Therefore, WM samples present more challenges to an algorithm, which has to perform in the conditions of reduced spatial resolution. In particular, the algorithm has to be able to resolve overlapping objects.

**Figure 6:**
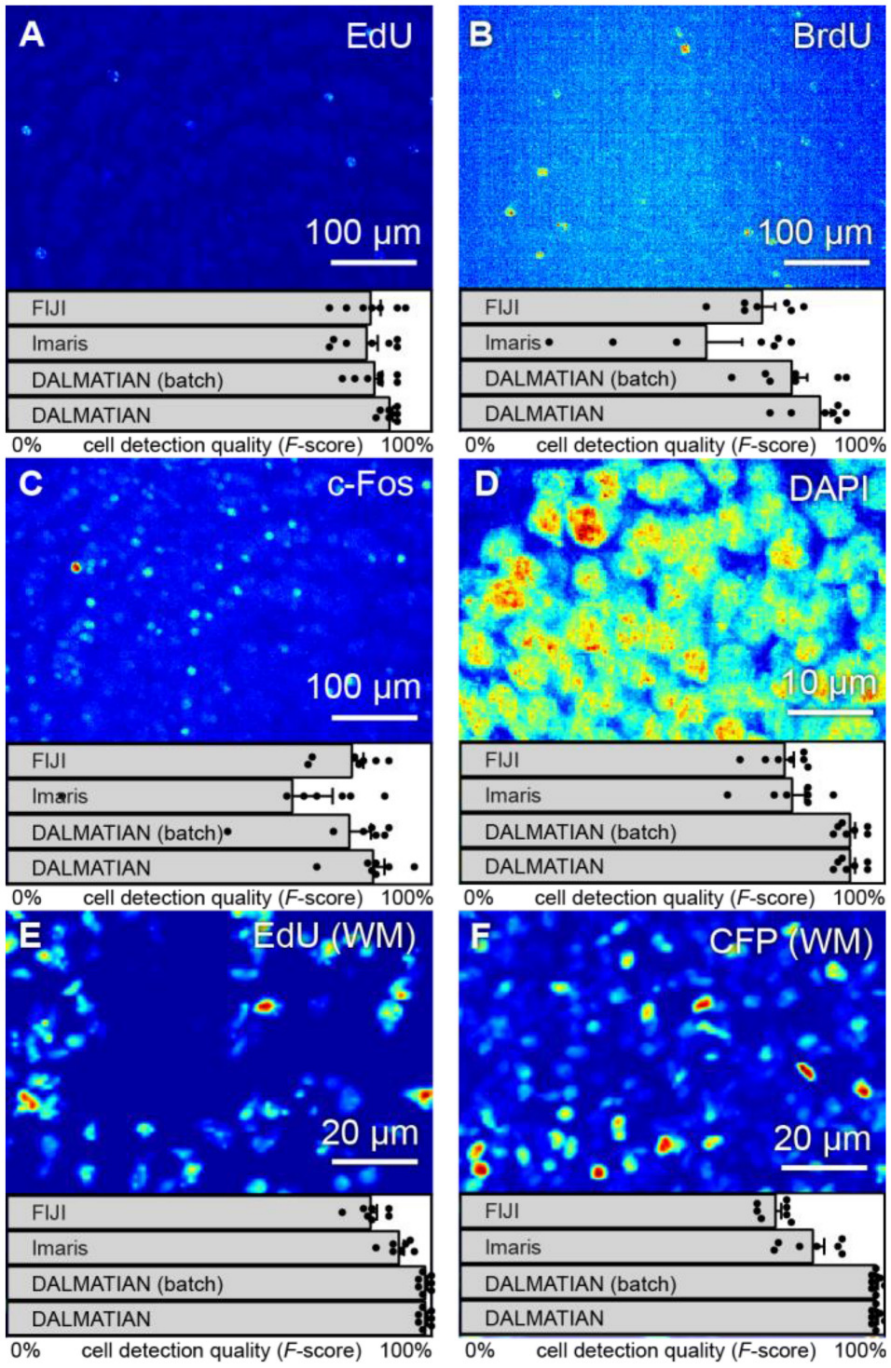
Various types of 3D samples (maximum intensity projections) and cell detection quality (*F*-scores). Tissue sections stained with **(A)** EdU, **(B)** BrdU, **(C)** c-Fos, **(D)** DAPI, **(E)** whole-mount hippocampi stained with EdU, and **(F)** nestin-CFPnuc.

To show how our algorithm deals with overlapping cells, we tested it on 3D images of EdU+ and CFP+ cells in WM samples (Figures 6E,F). CFP here stains neural progenitor cells—a densely packed cell population. EdU stained cells during cell division, which may not have enough time to move apart.

Cell detection quality (*F*-scores) provided by our algorithm is 99 ± 1% for EdU (WM) and 97 ± 2% for CFP (WM). These high values reflect high SNR in the samples used (16.9 and 10.7 dB, respectively). FIJI provided cell detection quality of 86 ± 4% for EdU (WM) and 74 ± 4% for CFP (WM), whereas Imaris provided cell detection quality of 93 ± 3% for EdU (WM) and 83 ± 7% for CFP (WM) on the same set of samples (all measures are in *F*-scores). Runtime with our algorithm for a whole mount hippocampus sample (3,000 × 3,000 × 300 pixels) was roughly 1 h using a computer server (4-Xeon Supermicro server with 32 GB of RAM). Notably, runtime and memory usage may be reduced with optimization and parallel computations. Therefore, our algorithm can deal with the issues specific to WM samples and provided the best cell detection quality (with regard to human experts) compared to the other automatic algorithms.

### Our algorithm is not limited to a particular resolution

Data resolution may vary across samples due to staining type, microscope used, sample volume and the desired level of detail. Therefore, cell detection algorithm should allow switching between data resolutions. To apply our algorithm to datasets with different resolution, one needs to scale the spatial parameters accordingly. The parameters (low-pass and high-pass filter standard deviations, cell size ranges) can be set independently for each axis, which makes it applicable to anisotropic resolutions. In particular, our algorithm allows the user to have the different parameters for the Gaussian in different directions.

To show how our algorithm addresses different resolutions, we provide cell detection qualities for EdU cells in WM samples, acquired with lower resolution (axial 7 μm, lateral 2 μm), and in tissue sections, acquired with higher resolution (axial 0.5 μm, lateral 1 μm) (Figures 6A,E). Both types of the samples featured high cell detection quality (*F*-score): 99 ± 1 and 84 ± 2%, respectively. Higher cell detection quality (*F*-score) in samples with lower resolution can be explained by a larger SNR in these samples (16.9 and 6.2 dB, respectively). Thus, our algorithm can deal with datasets obtained at different imaging resolutions.

### Our algorithm allows for batch cell detection

Cell detection in batches is important for increasing throughput, as it alleviates the necessity to tune the detection parameters for each individual sample. This seemingly easy task is not trivial because of sufficient differences between samples. To show how our algorithm addresses this issue, we present the batch testing results for all the samples under study (Figure 6). All the parameters were constant for each sample type.

Batch cell detection quality provided by our algorithm is 86 ± 5% for EdU, 78 ± 9% for BrdU, 81 ± 13% for c-Fos, 92 ± 3% for DAPI, 86 ± 2% for EdU (WM) and 57 ± 9% for CFP (WM) (all measures are in *F*-scores). Importantly, when compared to human expert, cell detection quality in batches of samples provided by our algorithm exceeded two other software packages (Imaris and FIJI) with individual settings for each sample. Thus, we consider our algorithm reliable in batch cell detection.

### Dependence of cell detection quality on noise

**Figure 7:**
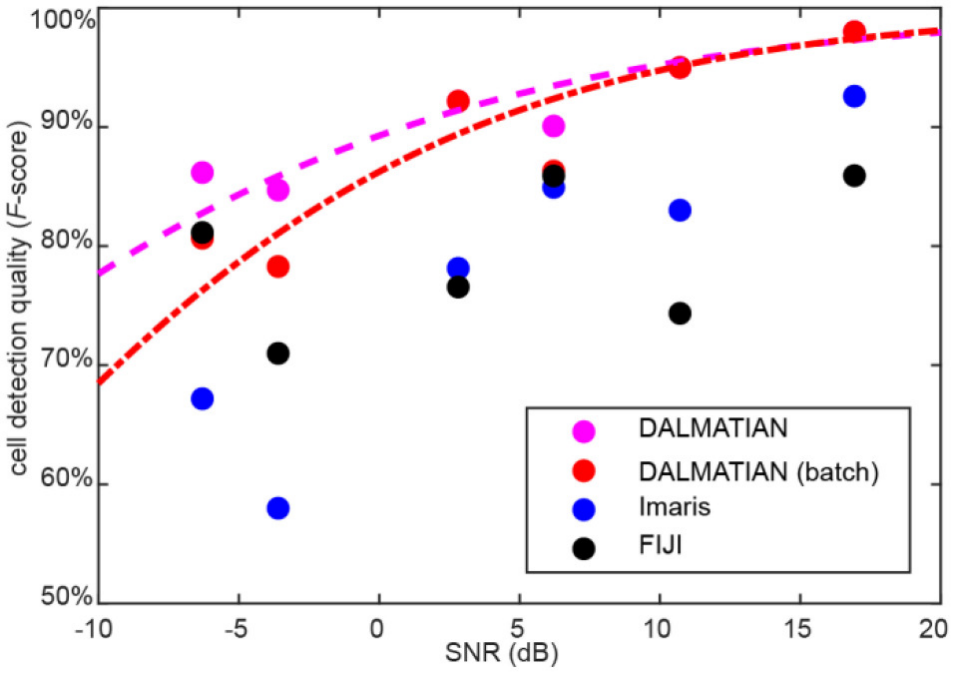
Dependence of cell detection quality on the SNR. Sigmoid fits for our algorithm are provided.

One limitation of our algorithm is that cell detection quality decreases for noisy data. To quantify this effect, we measured SNR for every sample under study. We defined SNR as 20 logarithms of signal amplitude to noise amplitude ratio. SNR defined like this is measured in decibels (dB). Average signal amplitude was measured as a difference between signal and background, whereas average noise amplitude was measured as a standard deviation of the data after high-pass filtering. For every sample type, we plotted the average cell detection quality (*F*-score), as a function of the average SNR. To compare between different software, we show the data for our algorithm, Imaris and FIJI. The data suggests that despite of the decrease of cell detection quality (*F*-score), at lower SNR values, our algorithm provides the best cell detection quality among the tested software (Figure 7).

## Discussion

Since 1960s, when the first systems were developed to automate cell detection in images (Meijering, 2012), a number of cell detection algorithms has been published. Most of these algorithms share the common approaches (Wu et al., 2008; Meijering, 2012), including intensity thresholding (Otsu, 1975; Jones et al., 2005; Xiong et al., 2006), feature detection (Meyer, 1979; Lindeberg, 1994; Demandolx and Davoust, 1997), morphological filtering (Wu et al., 2008), region accumulation (Malpica et al., 1997) and deformable model fitting (Kobatake and Hashimoto, 1999; Esteves et al., 2012). Cell detection algorithms would usually combine these approaches to overcome its individual limitations (Meijering, 2012), and claim the superior performance in specific tasks (Meijering, 2012). Therefore, there is a demand for a cell detection algorithm generic enough to be easily adjustable for a wide range of applications (Schmitz et al., 2014). To address that demand, we started with building a list of challenges for the automatic algorithms of cell detection. These challanges included differences between samples, autofluorescence, inhomogeneous staining, varying background, and overlapping cells. We developed and implemented a new algorithm for 3D cell detection, keeping these challenges in mind to be able to deal with a wide range of sample types.

We used histogram equalization for the reduction of autofluorescence and to provide the ability of batch detection with a single set of parameters. Gaussian 3D low-pass filtering was successful in dealing with inhomogeneous staining. Gaussian 3D high-pass filtering with subsequent thresholding was efficient in reducing varying background. A watershed procedure is effective in splitting images into segments, containing zero or one cell. A bootstrap fitting procedure was effective in establishing the statistical significance of the watershed segments as cells. We showed that one set of parameters was sufficient for handling samples of the same type in a batch mode. Tests performed on 42 samples, representing 6 different staining and imaging techniques, have shown that our algorithm enables reliable detection of cells of different brightness, non-uniformly stained and overlapping cells in whole brain regions and individual tissue sections. The comparison to human expert annotations have shown a favorable performance described by the range of *F*-scores between 78 and 99% (Figure 7). These scores exceeded results obtained with other software packages, such as FIJI and Imaris. Thus, our algorithm has addressed the typical challenges of automatic cell detection and yielded high cell detection quality. One factor to our knowledge that impaired cell detection quality was SNR. Testing on various types of the samples shows that our algorithm is generic enough to be adjustable for a wide range of applications. The software is available at http://github.com/koulakovlab/dalmatian.

## Author contributions

All authors listed have made a substantial, direct and intellectual contribution to the work, and approved it for publication.

### Conflict of interest statement

The authors declare that the research was conducted in the absence of any commercial or financial relationships that could be construed as a potential conflict of interest.
